# Ultrasound-Guided Erector Spinae Plane Block: A Case Series Demonstrating Utility for Acutely Painful Conditions in the Emergency Setting

**DOI:** 10.7759/cureus.67327

**Published:** 2024-08-20

**Authors:** Richard Slama, Julia Lerner, Adrianna Kyle

**Affiliations:** 1 Emergency Medicine, Riverside Regional Medical Center, Newport News, USA

**Keywords:** pocus, fracture, emergency medicine, ultrasound, regional anesthesia

## Abstract

Ultrasound-guided erector spinae plane block (ESPB) has emerged as a valuable technique in pain management. Though frequently used in chronic and postoperative pain, it remains underutilized in the emergency department (ED) setting. In particular, this block has become attractive because it is rapid, safe, and efficacious for a variety of different pain syndromes that are commonly encountered in the emergency department setting. Of particular importance is that this block results in pure sensory blockade, allowing patient movement after the procedure has been performed. This case series explores the efficacy of the ESPB in various clinical scenarios, including refractory cervical radiculopathy, rib fractures, obstructive nephrolithiasis, and sciatica. Each of these patients presented with symptoms of refractory aggressive pain management strategies, including non-steroidal anti-inflammatory drugs (NSAIDS), acetaminophen, narcotics, muscle relaxers, and ketamine. After undergoing ESPB, the patients were able to be successfully discharged without return visits to the emergency department for the return of their pain. This case series aims to show the utility of this procedure for refractory painful conditions and also reviews other indications where the block may be used. While previous reports have shown the utility of this block at individual levels, we present the flexibility of this block being used at multiple levels of the spine.

## Introduction

The erector spinae plane block (ESPB) was first used in 2016 as a means of controlling post-operative neuropathic pain in the thoracic region [1,2]. Since that time, it has rapidly evolved into a multifaceted plane block with applications from the cervical spine to the sacrum [3,4]. The most common method for performing this is an in-plane technique under ultrasound guidance. Although the exact mechanism of the block remains incompletely understood, proposed mechanisms suggest its spread to the ventral rami, dorsal rami, paravertebral area, and even the epidural space [5]. Additionally, this block provides visceral coverage by way of the rami communicantes [6]. This block extends to multiple dermatomal levels in a single injection, leading to larger areas of local anesthesia. In the thoracic area, each 3.3 mL of injected local anesthetic provides an additional dermatomal level of coverage [7]. Additionally, this block is associated with fewer adverse outcomes than other commonly performed truncal blocks because they are farther from critical structures such as the pleural or epidural space [8]. Some rare complications, such as transient motor weakness and harlequin syndrome, have been described in case reports, but the most common complications are infection, local anesthetic toxicity, vascular puncture, pleural injury, and pneumothorax [9-11]. The exact rate of complications, while not well established, is thought to be extremely low.

There is no doubt that the healthcare system is facing an unprecedented challenge of worldwide opioid misuse and abuse [12-15]. Emergency physicians are well situated to help combat this issue, as acute and chronic pain are commonly presenting issues [16]. More importantly, poorly treated acute pain may lead to the development of chronic pain [17]. Treatment of chronic pain with opioids is not recommended by multiple guidelines, and the receipt of opioids for chronic pain can induce a vicious cycle of opioid-induced hyperalgesia [18,19]. The importance of the use of alternatives for pain management has been previously highlighted by other authors, but opioids are still being used and prescribed for conditions with little or no supporting evidence [20]. With the increased implementation of ultrasound training in residency programs, regional anesthetic techniques are becoming a more viable option in the management of pain. Ultrasound guidance substantially improves these procedures' efficacy, efficiency, and safety. The American College of Emergency Physicians currently considers US-guided regional anesthesia to be a core competency of emergency physicians [21]. While many regional anesthetic techniques can risk neuro-vascular injury, ESPB is a plane block with an extremely low incidence of complications. The block is easily taught because the son anatomy is relatively straightforward, critical structures are generally absent, and there is a bony backstop, that serves as the endpoint for the block.

The goal of our investigation is to present a series of cases in which patients with refractory pain were treated using the ESPB. Of significant importance, we highlight the uses of this block for a variety of conditions. In each of these cases, this technique was found to be a safe, multifaceted, easily performed procedure where traditional methods for pain control had failed.

## Case presentation

Four cases are presented, each demonstrating the successful application of ESPB in the ED setting. The technique involved the injection of local anesthetic in the fascial plane between the erector spinae muscles and the transverse processes of the vertebrae, providing targeted analgesia to the dorsal and ventral rami of the ipsilateral spinal nerves. ESPB induces a purely sensory block in a dermatomal distribution without causing motor impairment.

**Figure 1:**
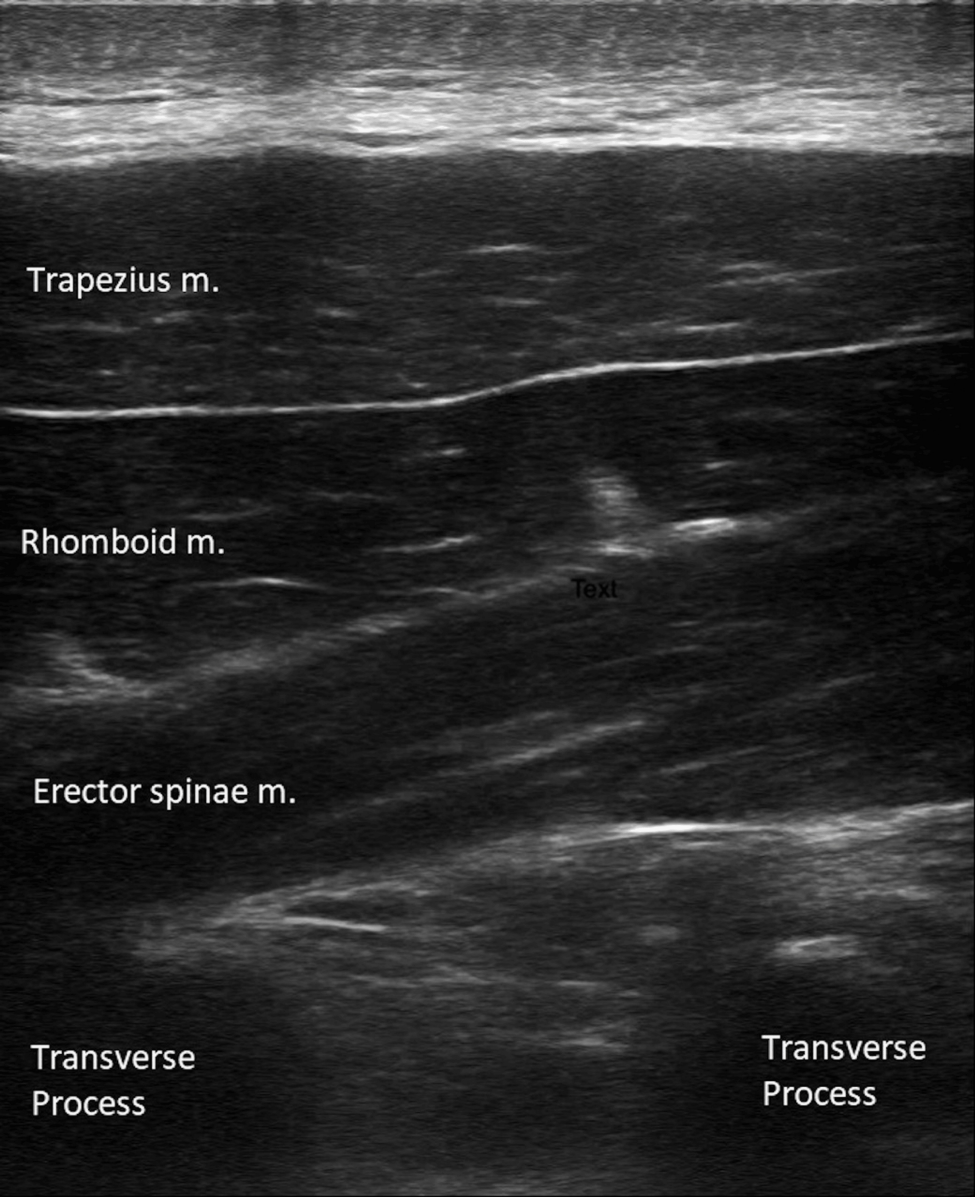
Ultrasound anatomy of the erector spinae plane block This is an original image produced by the authors of the paper.

With the exception of the lumbar spine and morbidly obese patients, the authors use a linear probe to perform this procedure. The level of the block is determined by the level of the injury or the corresponding dermatome where the painful condition exists. In the posterior thoracic area, the first rib can be identified using ultrasound, and the ribs can be counted caudally until the appropriate level is determined. At this point, the proceduralist can trace the rib medially to identify the corresponding transverse process. The level is then marked, and the block may be performed. In the upper thoracic area, the anatomy is easily recognizable using ultrasound to visualize the trapezius, rhomboid, and erector spinae muscles overlying the transverse process (Figure 1). Moving laterally to medially, the identification of the transverse process is confirmed as an isolated flat hyperechoic structure with frequent absence of the pleura. The ribs, by comparison, are more rounded, and the pleura is readily identified underneath. Once the correct view is obtained, a needle is advanced in the plane to contact the transverse process, and local anesthetic should be seen spreading along a distinct fascial plane under the muscle.

Case 1: cervical radiculopathy 

**Figure 2:**
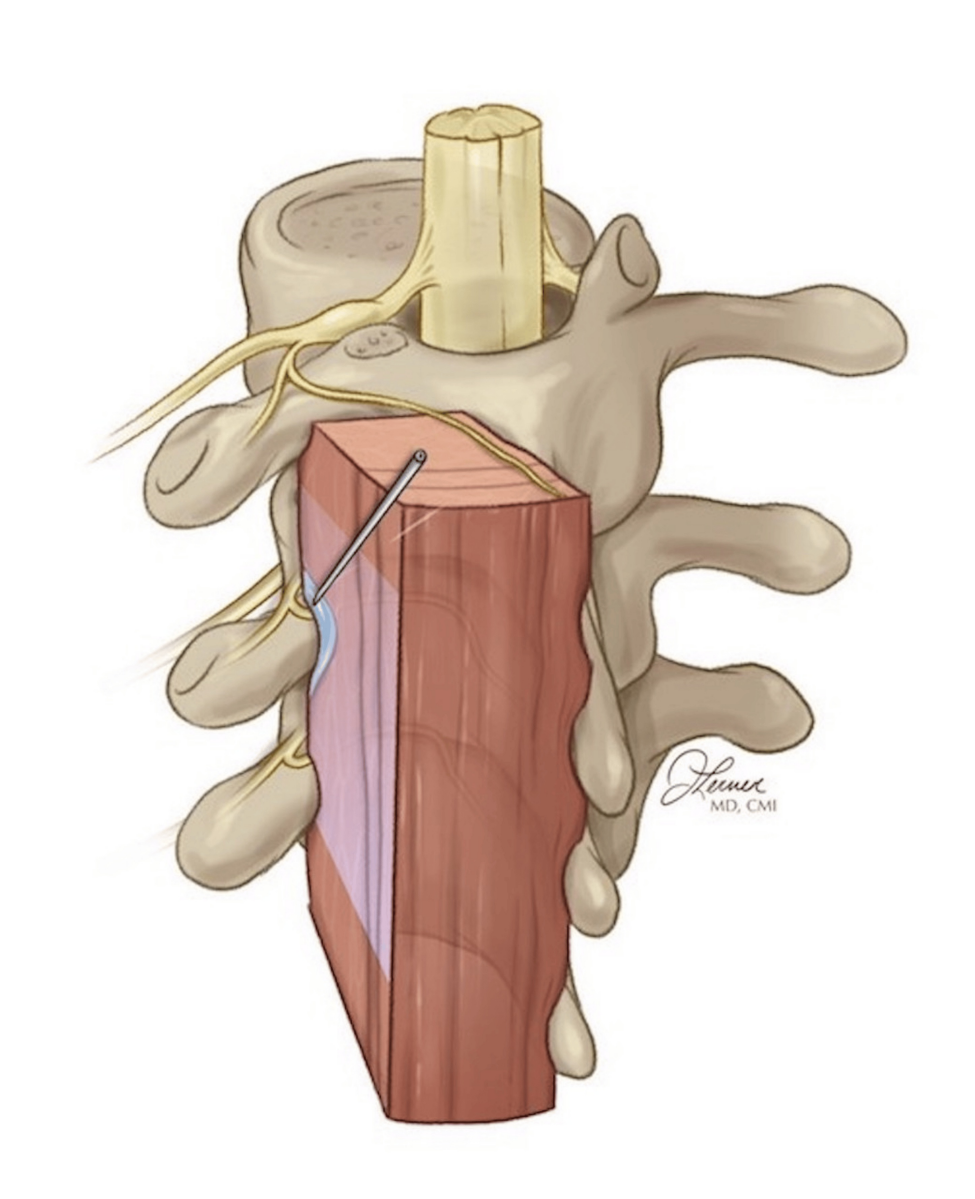
Sagittal view of the erector spinae plane block Notice that the needle is directed at the transverse process, and the local anesthetic is spreading along the fascial plane. These images are original illustrations of the author (JL) and have been utilized with her permission.

A 52-year-old patient presented for the second time in 24 hours with acute to chronic 10/10 pain of left-sided cervical radiculopathy refractory to non-steroidal anti-inflammatory drugs (NSAIDS), narcotics, gabapentin, and muscle relaxers. His medical history was significant for well-controlled HIV as well as hypertension. Vital signs were within normal limits, with the exception of hypertension, at 152/93. His physical exam was significant for left cervical paraspinal tenderness to palpation, a positive spurling sign, and no neurological deficits. Using 20 mL of 0.25% ropivacaine, a T2 ultrasound-guided ESPB led to rapid pain relief, allowing for improved range of motion and patient comfort (Figure 2). The patient reported significant alleviation of pain to 3/10 at a 72-hour telephone follow-up and was able to manage his pain with medications in his home. The patient then followed up with his primary care physician for further care and management. This case illustrates the value of this technique in pain cycle interruption when multimodal pain control has failed. In this particular case, the authors do not recommend an injection of more cephalad than T1, as critical structures such as the vertebral arteries, epidural space, and other neurovascular structures tend to lie more superficially in this region. This case highlights the utility of this technique in pain cycle interruption.

Case 2: rib fractures 

**Figure 3:**
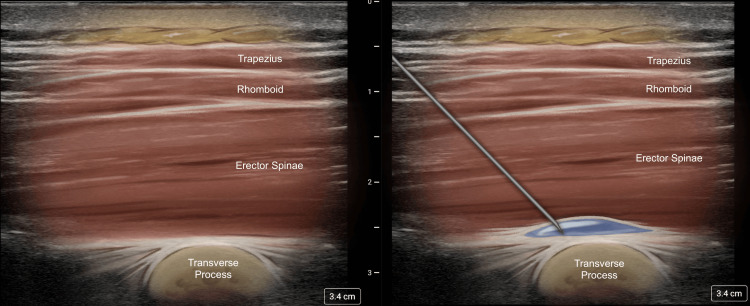
Illustrated ultrasound anatomy before and after erector spinae plane block All images are original illustrations used with permission from the author (JL).

A 71-year-old patient with a history of hypertension and diabetes presented fractures of the left posterior sixth and seventh ribs after a mechanical fall. The patient was experiencing severe 10/10 pain that was not responding well to acetaminophen, NSAIDS, muscle relaxers, lidocaine patches, and narcotics. He was noted to be hypertensive to 167/99 and tachycardic to 115, with an O_2_ saturation of 95% and a respiratory rate of 25. Using 30 mL of 0.5% ropivacaine, a T5 ESPB effectively alleviated pain to a 2/10, and his vital signs normalized after treatment. The patient was discharged with incentive spirometry, multimodal pain control, and rescue narcotics. In this case, the patient received 8 mg of intravenous dexamethasone for block prolongation. The patient did not require a return visit to the ED for admission or respiratory compromise. Figure 3 illustrates the expected ultrasound appearance at the T5 level, both before and after the injection. Please note that the goal is to lift the erector spinae muscle off the transverse process as opposed to injecting it within the muscle. The authors have found it most useful to aim for the corner of the transverse process to accomplish this while avoiding puncturing deep into the inter-transverse space. This case highlights how the ESPB can be useful in the treatment of acute traumatic conditions.

Case 3: refractory nephrolithiasis pain 

**Figure 4:**
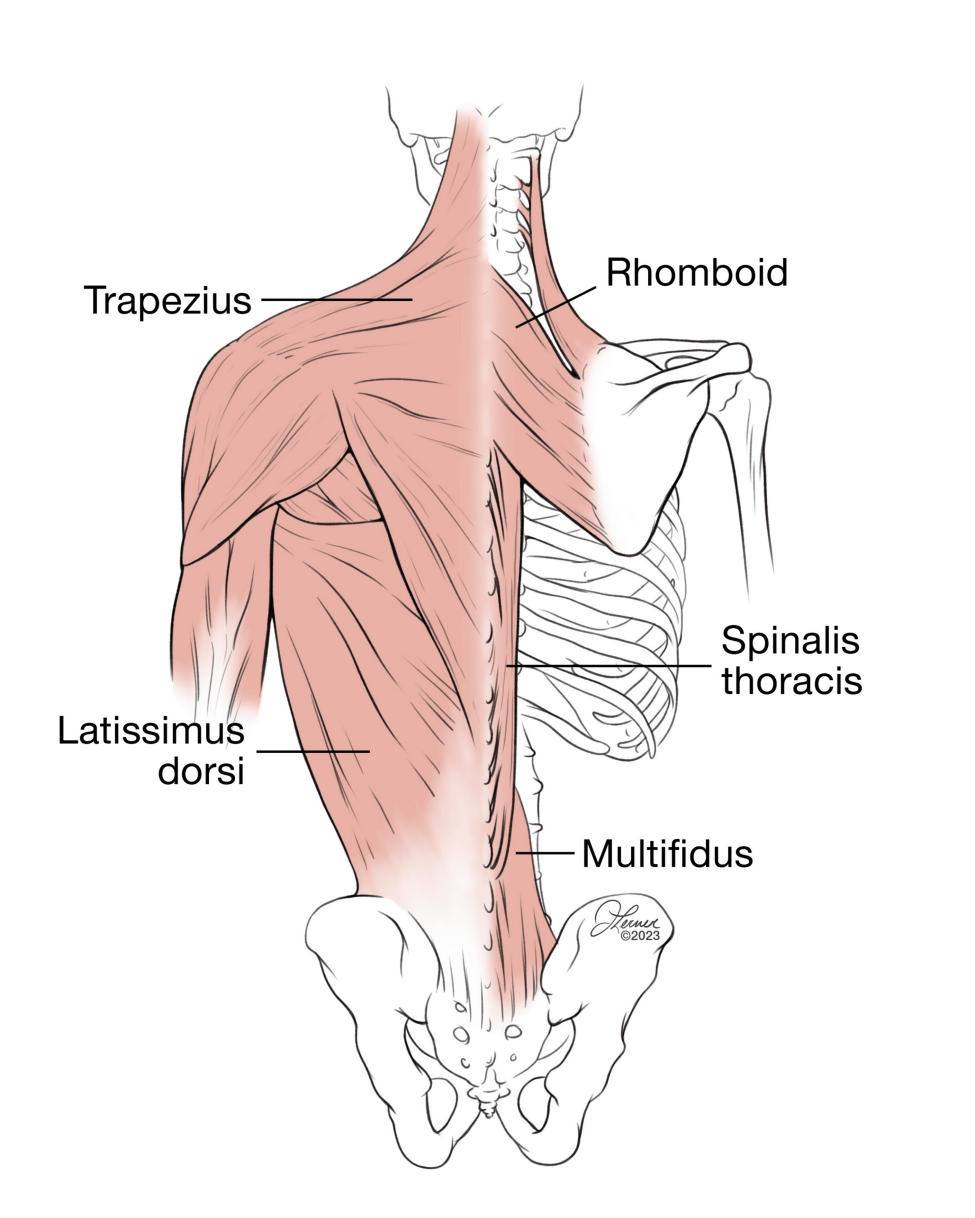
Anatomy of the paraspinal musculature All images are original illustrations used with permission from the author (JL).

A 51-year-old patient with a history of diabetes, hypertension, and nephrolithiasis presented with a displaced percutaneous nephrostomy tube and obstructive nephrolithiasis. Her vital signs, with the exception of tachycardia to 120 were within normal limits. While awaiting tube replacement, the patient was experiencing 10/10 severe pains refractory to NSAIDs, intravenous opioids, and ketamine. The patient underwent ultrasound-guided T8 ESPB with 30 mL of 0.25% ropivacaine, resulting in significant pain alleviation to a 1/10 on the pain scale. This relief persisted until she underwent the replacement of a percutaneous nephrostomy tube without incident eight hours later. This case illustrates an interesting aspect of this block providing visceral pain relief, likely via the rami communicantes. The anatomy of the block can be visualized in Figure 4.

Case 4: refractory sciatic pain 

A 50-year-old patient with a history of sciatica presented with refractory left-sided sciatic pain refractory to treatment with NSAIDS, narcotics, acetaminophen, gabapentin, and steroids. He had been seen numerous times over the past month by his physician for the pain, and despite escalating treatments, it had gradually become more intense and severe. He was on chronic opioid therapy (oxycodone 10 mg) for this pain. His physical exam was significant for a positive straight leg raise test on the left side, but there were no neurological deficits. When palpating the patient's left hamstring and moving his lower extremity, he experienced a painful stimulus that was out of proportion to his examination. Despite all of the aforementioned treatments, his pain was still rated as a 9/10, and he was unable to move from lying to standing. He was felt to be experiencing opioid-induced hyperalgesia and underwent treatment with 30 mg IV ketamine in the ED but was still unable to ambulate due to pain. An ultrasound-guided L3 ESPB with 30 mL of 0.5% ropivacaine was performed, resulting in substantial pain reduction to a 4/10. The patient was able to ambulate without issue and did not require a return visit for pain control or admission. The purely sensory block allowed the patient to ambulate without motor symptoms, emphasizing its potential for outpatient pain management. Opioid-induced hyperalgesia can make the management of exacerbations of chronic pain exceedingly difficult with traditional approaches. This case demonstrates the adjunctive use of this technique for a patient where other treatments have failed.

**Figure 5:**
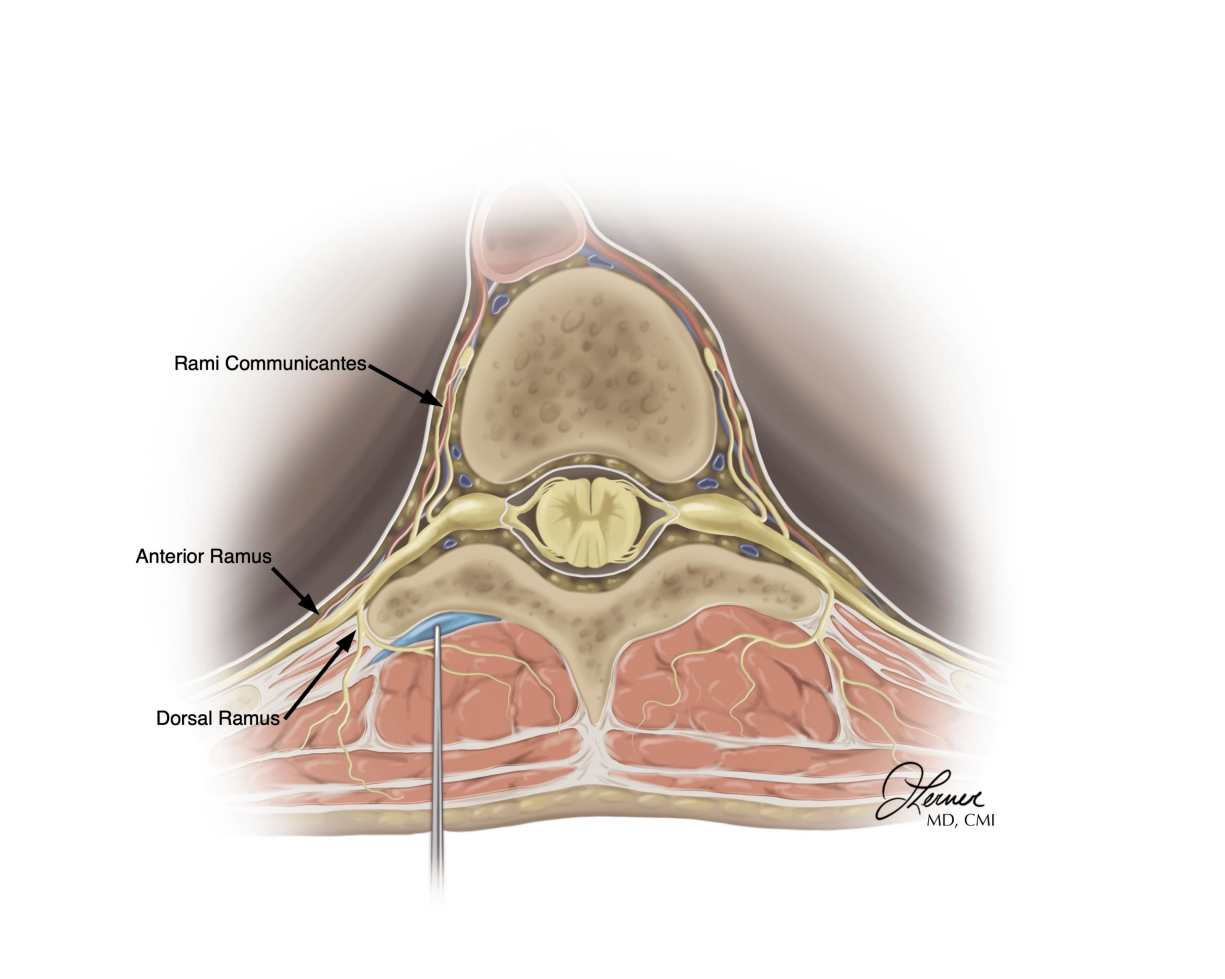
Axial view of the erector spinae plane block Notice that the needle is directed over the transverse process. Note the anatomy of the dorsal ramus, ventral ramus, and rami communicantes. These images are original illustrations of the author (JL) and have been utilized with her permission.

The lumbar-level ESPB can be technically more challenging for multiple reasons. Due to the depth of the transverse processes at this level, the curvilinear probe is best suited to perform this block. To ensure that the appropriate level is achieved, the sacrum is first identified, and the probe is moved to the cephalad, counting the vertebrae and marking them at the appropriate level. The probe is then used to identify the more superficial spinous process, then marched laterally until the bony target disappeared, and then brought back medially to re-identify the lateral aspect of the transverse process. Care must be taken at this level to avoid injection into the more medial retrolaminar space or, laterally, missing the transverse process altogether. Usually, the angle of approach is more acute, so careful needle tracking and hydrolocation are important. Please note that when performing the erector spinae plane block at the level of the lumbar spine, the trapezius, and rhomboid muscles are not present. Only the erector spinae muscle can be seen overlying the transverse process (Figure 5).

## Discussion

The ultrasound-guided ESPB represents a promising addition to the repertoire of physicians managing diverse pain presentations. This case series and accompanying illustrated technique demonstrate the ease and efficacy of ESPB for providing targeted analgesia in myriad scenarios while allowing patients to maintain motor function completely. While previous reports have highlighted the use of the ESPB in a wide variety of clinical scenarios, this series combines the utility of this block for painful exacerbations of acute flares of chronic pain, acute traumatic conditions, viscerally painful conditions, and as an adjunct for opioid-induced hyperalgesia. We believe this is the first series that successfully describes the implementation of this block at each anatomic level for a myriad of conditions without complications or ED bounce back for the return of pain.

When performing this block, there are various considerations, including the laterality, the level being performed, and the condition desired to be treated. ESPB can be performed bilaterally if needed, but this can limit the volume of local anesthesia given. In general, every 3.3 mL of injected local anesthetic will provide an additional dermatomal level when averaged over the entirety of the spine. However, the thoracic region requires less local anesthesia (2.5 mL) when compared to the lumbar spine (5 mL) for each dermatomal level [22]. The concentration of the local anesthesia is also important when performing this block. In general, we prefer ropivacaine as it is less cardiotoxic and tends to provide more selective sensory blockade when compared to bupivacaine [23]. When performing this block, we found the highest level of pain relief in rib fractures, nephrolithiasis, and low back pain using 30 mL of 0.5% ropivicaine. In frail patients or patients whose weight does not allow for higher concentrations, we will dilute the ropivacaine to 0.25-0.375%. When used at the T2 level, this technique has previously been described when other modalities have failed or are contraindicated [24].

Radiculopathy and neuropathic pain are frequent complaints in the ED, and often, these patients have already received multiple medications for their symptoms. Current recommendations for the management of this condition include NSAIDS, muscle relaxers, and steroids [25]. In many cases, these patients are then seen in the ED, where they are then treated with narcotics. The evidence for the use of narcotics for radicular or neuropathic pain is weak, and repeated use can lead to states of opioid-induced hyperalgesia. Further inadequately treated pain can lead to a phenomenon known as ” winding,” where pain can be increased in a frequency-dependent manner [26]. This phenomenon may also be involved in pain sensitization or the development of chronic pain [27]. Use in shoulder and proximal humerus surgeries has also been described when sparing of the phrenic nerve is of concern [28-30]. Theoretically, this block could be used as a bridge to surgery for trauma patients awaiting definitive management of their shoulders or proximal humerus. Harlequin syndrome is a particularly important risk to discuss with the patient before performing the procedure at this level. This syndrome is unilateral hemifacial anhidrosis and vasoconstriction, which is due to the inadvertent blockade of the T2-T3 sympathetic fibers. If this does occur, it is usually self-resolving and does not require further treatment. The authors of this paper do not advocate performing this block above T1, as there are multiple important anatomic structures above this level, including the vertebral arteries.

Rib fractures, in particular posterior rib fractures, have previously provided challenges for pain control. When performed at the level of T4, the spread of local anesthetics can cover a broad range of dermatomes and significantly improve pain. In previously performed studies, there have been positive effects on pain scores and incentive spirometry volumes [31]. When used at the level of T8, there have been numerous reports of use for renal colic, pancreatitis, biliary obstruction, and even abdominal malignancy [32,33]. When used at this level, it can also be implemented for some lower-occurring rib fractures. Complications at the thoracic level are rare and are usually limited to pneumothorax or thoracic cavity punctures.

When implemented at the level of L1, the ESPB can be used for lower quadrant abdominal pain. Of greatest interest is the visceral coverage that this block can provide for the often painful conditions that are present in the ED. In a case report, this block has been described as alleviating pain from appendicitis when used at this level. While we could not find described cases of diverticulitis or ovarian pathology, it is feasible that this block could not be used for these indications. Use at L4 can be implemented for sciatica, low back pain, and even hip fractures [34-36]. As displayed in Figure 5, the only muscles overlying the transverse process are the erector spinae muscles. Although complications at this level are rare, previous descriptions have documented cases of inadvertent lumbar plexus blockade.

As illustrated in Table 1, there are a multitude of different proposed uses for this technique. Of all those listed, the most well-studied and widely accepted use for these in the ED setting is for rib fractures. However, there have been many cases where this modality has proven effective in cases where traditional treatments have failed or been inadequate (Figure 1).

**Table 1:** Erector spinae plane block indications

Level	Proposed use	Volume (mL)	Ropivacaine Concentration (%)
T2	Cervical radiculopathy, shoulder injuries, and proximal humerus fractures	25	0.25–0.5
T4	Rib fractures, chest tube placement [37],and herpes zoster [38]	30	0.25–0.5
T8	Rib fractures [39], nephrolithiasis [33], cholecystitis, pancreatitis [40], and malignancy [41]	30	0.25–0.5
L1	Appendicitis [42,43] and diverticulitis, ovarian pathology	30	0.25–0.5
L4	Hip fractures [36] and low back pain [34]	30	0.25–0.5

## Conclusions

This regional technique is relatively new, and there are few RCTs in the ED setting assessing its true efficacy for various clinical conditions. The use of this block is relatively new, and most of the literature is case series or reports. While technically safer than other techniques, this block is not without risks, and the provider using this technique must weigh these potential risks against the benefits when performing the procedure. While RCTs have not been widely performed on this novel block, they have been shown to be useful in multiple scenarios where traditional methods may fall short. Given the safety of this block, ease of use, and versatility, it is a block that can widen the pain control armamentarium of emergency physicians. Further research is warranted to elucidate the full extent of the utility of ESPB in an emergency setting.
